# Analysis of Stress Relaxation in Bulk and Porous Ultra-High Molecular Weight Polyethylene (UHMWPE)

**DOI:** 10.3390/polym14245374

**Published:** 2022-12-08

**Authors:** Eugene S. Statnik, Alexey I. Salimon, Yulia E. Gorshkova, Natallia S. Kaladzinskaya, Ludmila V. Markova, Alexander M. Korsunsky

**Affiliations:** 1 HSM Laboratory, Center for Digital Engineering, Skoltech, 121205 Moscow, Russia; 2 “Luch” Laboratory, National University of Science and Technology MISiS, 119049 Moscow, Russia; 3 Frank Laboratory of Neutron Physics, Joint Institute for Nuclear Research, 141980 Dubna, Russia; 4 Institute of Physics, Kazan Federal University, 420008 Kazan, Russia; 5 Laboratory of Electron Probe Analysis, Department of Materials Research and Testing, SSI O.V. Roman Powder Metallurgy Institute, 220005 Minsk, Belarus; 6 MBLEM, Department of Engineering Science, University of Oxford, Oxford OX1 3PJ, UK

**Keywords:** UHMWPE, relative density, porosity, stress relaxation, *operando* analysis, Prony series, X-ray tomography, small angle X-ray scattering (SAXS), Dyben 1.0 miniature 1 kN universal mechanical testing

## Abstract

The reported study was devoted to the investigation of viscoelastic behavior for solid and porous ultra-high molecular weight polyethylene (UHMWPE) under compression. The obtained experimental stress curves were interpreted using a two-term Prony series to represent the superposition of two coexisting activation processes corresponding to long molecular (~160 s) and short structural (~20 s) time scales, respectively, leading to good statistical correlation with the observations. In the case of porous polymer, the internal strain redistribution during relaxation was quantified using digital image correlation (DIC) analysis. The strongly inhomogeneous deformation of the porous polymer was found not to affect the relaxation times. To illustrate the possibility of generalizing the results to three dimensions, X-ray tomography was used to examine the porous structure relaxation at the macro- and micro-scale levels. DIC analysis revealed positive correlation between the applied force and relative density. The apparent stiffness variation for UHMWPE foams with mixed open and closed cells was described using a newly proposed three-term expression. Furthermore, in situ tensile loading and X-ray scattering study was applied for isotropic solid UHMWPE specimens to investigate the evolution of internal structure and orientation during drawing and stress relaxation in another loading mode.

## 1. Introduction

Ultra-high molecular weight polyethylene (UHMWPE) is a versatile thermoplastic polymer showing great potential in many engineering, biomedical, and energy applications [1]. Fine tuning of the catalytic synthesis and processing conditions allows varying the crystallinity and density of physical cross-links (tangles), bond interchange, and chain scission [2], whilst the adjustment of extrusion and drawing operations controls the entanglement [3] and governs the orientation of the crystalline phase [4], allowing to create materials of nominally identical chemical composition with an extraordinarily wide range of mechanical performance characteristics, e.g., elastic modulus may vary between 1 and 20 GPa, and tensile strength between 40 and 5000 MPa [5].

Additional opportunities to modify the mechanical performance are provided by the technologies able to create stiff self-reinforced composites [6,7] or, in contrast, compliant hybrids using 1D (e.g., electrospun nanofibers [8,9]), 2D (foils and films [10,11]), and 3D porous UHMWPE [12]. Non-continuous materials like non-woven mats, folded or perforated films or sponges may produce elastic modulus as low as units of MPa [13]. It is natural to consider such materials for applications where large strains or cyclic loading prevail (self-positioning and self-locking bone or cartilage implants, dampers etc.). The relaxation of stresses under permanent set strain is a process often present in the mechanical response of engineering structures and devices.

Surprisingly, to the best of the authors’ knowledge, there have been very few studies devoted to the clarification of the peculiarities of UHMWPE relaxation in the bulk or porous forms [14]. The authors recently addressed a number of issues related to the fabrication [15], mechanical response [16], and biocompatibility [17] of open-cell porous UHMWPE. The study of stress relaxation in UHMWPE is a continuation and expansion of research in this field.

Porous UHMWPE can be fabricated via thermal sintering of loose powder [18] or desalination of UHMWPE-table salt composites [19]. Close-cell or hierarchically structured bimodal porous UHMWPE sponges can be manufactured using the drying of UHMWPE-xylene gels in supercritical fluids [20].

From the fundamental point of view, the cardinal aspect of the stress relaxation phenomenon in semicrystalline polymers is the interplay between molecular (e.g., physical cross-links and entanglements) and structural (recovery of cell walls, trusses and nodes in porous structure) elemental relaxation mechanisms. It may be surmised that the difference in scales and mechanisms is likely to lead to distinct characteristic times and activation energies.

The stress relaxation experiments reported here were undertaken in the chamber of scanning electron microscopy (SEM) during compression loading using an SEM-compatible portable Deben 1 kN testing rig, with additional characterization performed using X-ray tomography. The digital image correlation (DIC) algorithm was applied to the series of high-resolution SEM images to map and monitor sample strains. The findings were used to extract the values of the elastic modulus from the stress-strain data at initial stages of compression. The characteristic times of stress relaxation were evaluated in relation to the porosity and apparent compression strain.

Two distinct (‘short’ and ‘long’) characteristic times of stress relaxation were deduced from experimental data interpretation. Somewhat unexpectedly, these times appear to be almost independent on the porosity and the applied compressive strain. Moreover, feasibility studies were performed to collect SAXS data from samples of UHMWPE subjected to in situ tensile loading. The results revealed internal rearrangement of lamellae representing the characteristic nanostructural units typical of semicrystalline polymers. It is worth noting that fundamentally the same two-time relaxation behavior was found to prevail both for the tensile samples made from isotropic and continuous UHMWPE and in the same porous material under compression. Namely, stress relaxation analysis revealed the presence of ‘short’ and ‘long’ characteristic times of similar magnitude of a few tens and hundreds of seconds, respectively. This observation suggests that the presence of micron-sized pores and the inhomogeneity of deformation at this scale do not alter the underlying molecular relaxation mechanisms that govern the material’s viscoelastic response.

In the light of these findings, perspectives for further investigations are considered, and the applicability of present results for the practical engineering of biomedical components and systems are discussed.

## 2. Materials and Methods

### 2.1. Sample Preparation

Porous UHMWPE rectangular plates were fabricated using the sacrificial rock salt dissolution procedure described in reference [15]. The variation of the salt to UHMWPE powder ratio was used to obtain foams with porosity in the range from 60% to 80% as measured using gravimetric analysis.

The fabrication procedure is briefly outlined as follows. First, rock salt (NaCl) and GUR 4120 UHMWPE powders (average particle size is 120 μm and average molecular weight is 4.7 × 10^6^ g/mol) were classified by size separately using a set of sieves using a vibratory shaker Analysette 3 Pro (Fritsch GmbH, Idar-Oberstein, Germany). Rock salt (fraction with the particle diameter in the range 150–200 μm) and UHMWPE (fraction with the particle diameter in the range 75–150 μm) fractions were mixed for 1 h using a planetary ball milling machine Pulverisette 5 (Fritsch GmbH, Idar-Oberstein, Germany). The prepared mixture was hot-compacted inside a rectangular press form of 10 mm × 80 mm × 50 mm dimensions (width, length, height). Finally, rock salt was removed via dissolution in 1 liter of distilled water with frequent water changes during 2 days.

Solid UHMWPE samples were prepared without rock salt addition to obtain monolithic isotropic UHMWPE plates as reference. Moreover, thin film samples were obtained by careful microtome sectioning from isotropic bulk polymer sample consolidated from powder for in situ tensile loading and SAXS measurements. 

The dimensions of final porous and non-porous samples were 15 mm × 15 mm × 8 mm and 15 mm × 15 mm × 4 mm, respectively.

### 2.2. X-ray Tomography

Tescan CoreTOM instrument (Tescan Orsay Holding, Brno, Czech Republic) at the SSI “Powder Metallurgy Institute”, Minsk, Belarus was used to visualize and investigate the microstructural features and statistical descriptors of the porous structure such as the type of porosity, connectivity and shape of cells. These geometrical features give a better understanding of the origins of the observed phenomena and allow to select the correct parameters for the Gibson–Ashby model of Young’s modulus estimation discussed below.

The experiment was carried out under the following acquisition conditions: source to detector distance of 850 mm, source to object rotation axis distance of 28 mm, cone beam mode with vertical rotation axis and continuous rotation (fly scan). X-ray source voltage was 30 kV, exposure time of 300 ms per projection and rotation angle from 0° to 360° with the step of ~0.17°. The detector pixel size was 0.3 mm and the equivalent voxel size was 10 μm. The reconstruction process was performed using the dedicated *Aquila* software v.1.18 (Ghent, Belgium) [21] and involved common artifact treatment methods, e.g., ring removal and beam hardening filters, iterative axis of rotation search and reconstruction. For visualization and statistics extraction purposes, *ORS Dragonfly* software v.2021.3.0.1087 (Montreal, QC, Canada) [22] was used.

### 2.3. In-SEM Compression Testing

The compression testing setup used was similar to that described in reference [16]. The Deben Microtest 1 kN (Deben UK Ltd., Woolpit, UK) tensile stage was mounted within the chamber and synchronized with SEM image acquisition using Tescan VEGA3 (Tescan Orsay Holding, Brno, Czech Republic). SEM image acquisition frequency was set to 0.25 fps. The traverse speed was set to constant displacement speed of 0.5 mm/min, so that ~33.3 μm step was recorded every 4 s. Each porous structure was compressed on maximum crosshead span of 10 mm. Images were captured under the following conditions: low vacuum atmosphere inside column with 20 Pa, high voltage of 30 kV, working distance of 28 mm, spot size of 560 nm, field of view of 6 mm, BSE regime.

**Figure 1:**
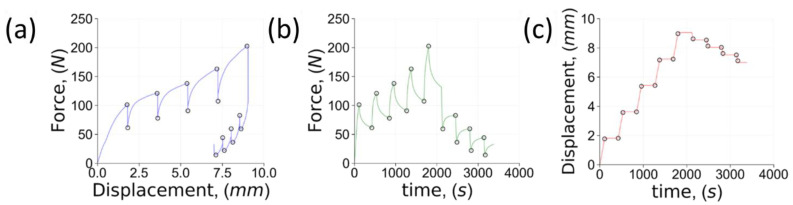
The specific protocol used for specimen compression and relaxation: (**a**) force-displacement curve; (**b**) time-dependent force and (**c**) time-dependent displacement. The black circles indicate the locations of relaxation start and finish.

The compression test was performed according to the protocol illustrated in Figure 1. After each incremental displacement ramp of 2 mm, the crosshead was stopped and the structure was allowed to relax for 5 min while the stress history was recorded. The step of 0.5 mm was used during unloading.

A different protocol was used for the isotropic bulk specimen due to the load capacity limit of the mechanical testing machine (maximum force). Hence, the same total number and duration of the loading steps was chosen, but the incremental displacement during loading was reduced to 0.5 mm from 2 mm.

**Figure 2:**
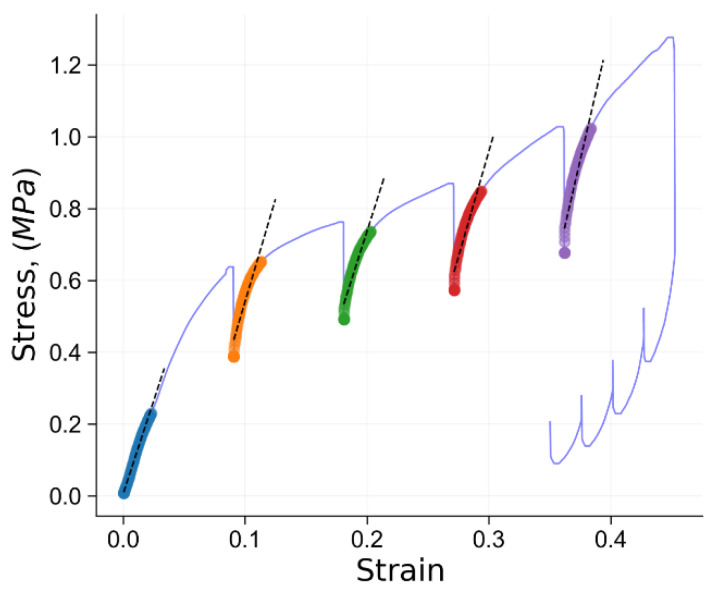
The schematic representation (black dashed lines) of the approximate locations of stiffness determination.

The initial loading stiffness of porous structures was calculated using the linear portion of the stress-strain curve during stress increase. Figure 2 provides a schematic indication of the locations and number of points from which the stiffness was calculated.

### 2.4. Porosity Evaluation

**Figure 3:**
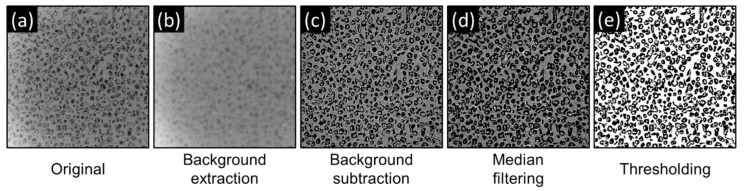
The porosity measurement protocol: (**a**) original image; (**b**) extracted image background; (**c**) B & C adjusted image after background subtraction; (**d**) median filtered image; (**e**) segmented image. The field of view of each image was kept constant at 6 mm × 6 mm.

The porosity changes during compression test were obtained from SEM images using *ImageJ* software v.1.8.0 (Bethesda, MD, USA) [23] and applying step-by-step protocol illustrated in Figure 3. The established protocol involves 4 basic steps, namely background extraction (rolling ball radius of 5 pixels with activated sliding paraboloid option) and subtraction to eliminate backlight bleeding effect; brightness and contrast adjustment; median filtering (radius of 3 pixels) and simple binary thresholding. Finally, the apparent porosity P is calculated according to the formula.

(1) $$P=\frac{A_{black}}{A_{overall}}$$

where $A_{black}$ is number of pixels with zero intensity and $A_{overall}$ is the total number of pixels. The apparent relative density can be simply defined as R=1−P.

### 2.5. Digital Image Correlation

**Figure 4:**
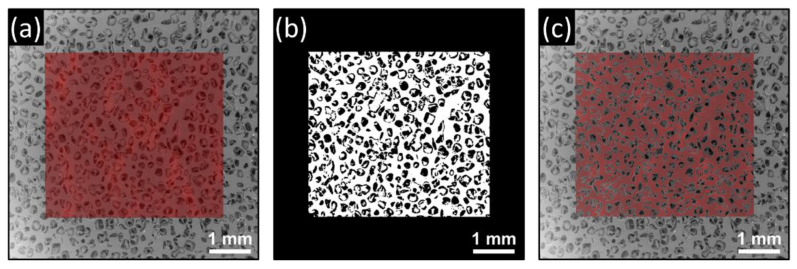
The adjustment procedure of obtained DIC analysis for porous structures: (**a**) initial appearance of structure with highlighted strain distribution; (**b**) mask creation stage: segmentation of SEM image and combination with region of interest used for DIC analysis; (**c**) result of applying mask for strain map.

The captured SEM images were post-processed using open-source 2D digital image correlation (DIC) software *Ncorr* v1.2 (Atlanta, GA, USA) [24]. Using the next set of parameters: subset radius of 30 pixels, subset spacing of 2, number of iterations 50, number of threads 12, “seed propagation” analysis with enabled auto propagation option. Next, the results of DIC analysis were corrected according to the procedure illustrated in Figure 4. The data obtained inside pores, i.e., empty spaces, were masked and removed from obtained strain maps.

### 2.6. Stress Relaxation

**Figure 5:**
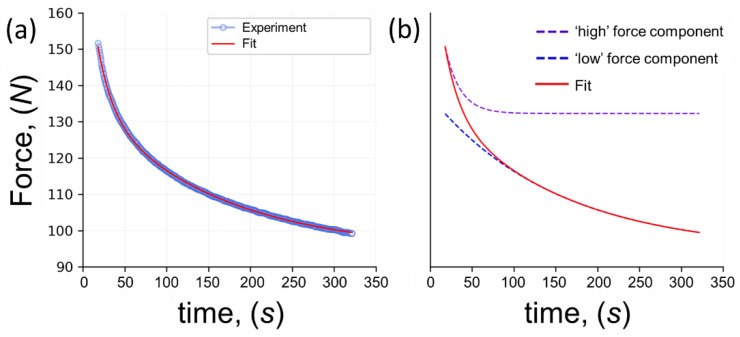
The illustration of the relaxation curve: (**a**) experimental data (semitransparent light blue circles) and fitting result (red curve); (**b**) decomposition of the fitted function (‘high’ force component is purple dashed curve while ‘low’ force component is blue dashed curve).

An example stress relaxation curve for UHMWPE foam is shown in Figure 5a. The obtained curves were fitted with a well-known Prony series representation [25]

(2) $$\sigma \left(t\right)=E_{\infty }\varepsilon \left(0\right)+\overset{N}{\underset{i=1}{\sum }}\sigma_{i0}\exp\left(-\frac{E_{i}}{\eta_{i}}t\right)=E_{\infty }\varepsilon \left(0\right)+\overset{N}{\underset{i=1}{\sum }}\sigma_{i0}\exp\left(-t/\tau_{i}\right),$$

where $E_{\infty }$ is the material equilibrium modulus,

$\varepsilon \left(0\right)$ is the accumulated strain up to the initial instant $\left(t=0\right)$,
$E_{i}$ are the values of Young’s moduli,
$\eta_{i}$ are viscosities,
and $\tau_{i}=\frac{\eta_{i}}{E_{i}}$ are relaxation times.

The parameters of $E_{i}$ and $\eta_{i}$ are either constant or slightly dependent on the strain ε at which relaxation is performed, but not dependent on time. The fitted function included only two terms due to better approximation according to the chi-square test for both short or ‘high’ force (purple dashed curve) and long or ‘low’ force (blue dashed curve) term behaviors simultaneously as shown in Figure 5b. Furthermore, $\sigma_{01}$ and $\sigma_{02}$ parameters are connected with the previous loading history while $\tau_{1}=\frac{\eta_{1}}{E_{1}}$, $\tau_{2}=\frac{\eta_{2}}{E_{2}}$, and $E_{\infty }$ parameters need to be considered as material characteristics based on the Maxwell definition.

### 2.7. In Situ Tensile Loading and X-ray Scattering Study

Xeuss 3.0 (Xenocs, Grenoble, France) laboratory X-ray beamline employed in this study is located at the Frank Laboratory of Neutron Physics of the Joint Institute for Nuclear Research (FLNP JINR, Dubna, Russia). The machine was operated using a Cu microfocus source at the voltage of 50 kV and current of 0.6 mA. The beam was conditioned to improve parallelism using multi-layer mirrors and collimated to the nominal cross-section of 0.15 mm × 0.15 mm. Scattering patterns were recorded using Eiger2 R 1 M detector (Dectris, Taefernweg, Baden, Switzerland) with 5 min exposure time per each 2D SAXS image acquisition at the detector-to-sample distance of 350 mm. SAXS data was interpreted by 1D correlation function analysis using Sasview 5.0.3 software (www.sasview.org (accessed on 7 October 2022)).

The application of controlled deformation within the vacuum chamber of the beamline was performed using the Dyben 1.0 miniature 1 kN universal mechanical testing device (HSM laboratory, Skolkovo Institute of Science and Technology, Moscow, Russia). The device allows controlled displacement of the crosshead with sample grips and continuous electronic recording of the load cell signal, as shown in Section 3 below. The experiments were performed using a constant traverse speed of 2 mm/min.

**Figure 6:**
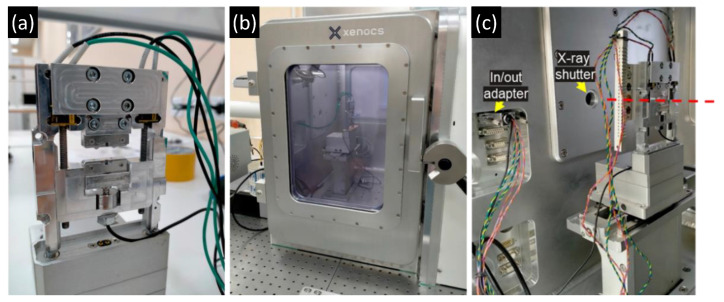
Illustration of the in situ SAXS tensile experiment set up: (**a**) the Dyben miniature 1 kN universal mechanical testing device attached to Xeuss 3.0 platform; (**b**,**c**) outer and inner views of evacuated Xeuss 3.0 chamber with Dyben 1 kN stage mounted.

The tensile specimens were extracted from a bar of isotropic UHMWPE and subjected to stepwise extension with pauses to record stress relaxation. At each step, the SAXS pattern was recorded in order to obtain insights concerning the nanostructural changes in the semicrystalline sample structure. The setup of the experiment is illustrated in Figure 6.

## 3. Results and Discussion

### 3.1. X-ray Tomography

**Figure 7:**
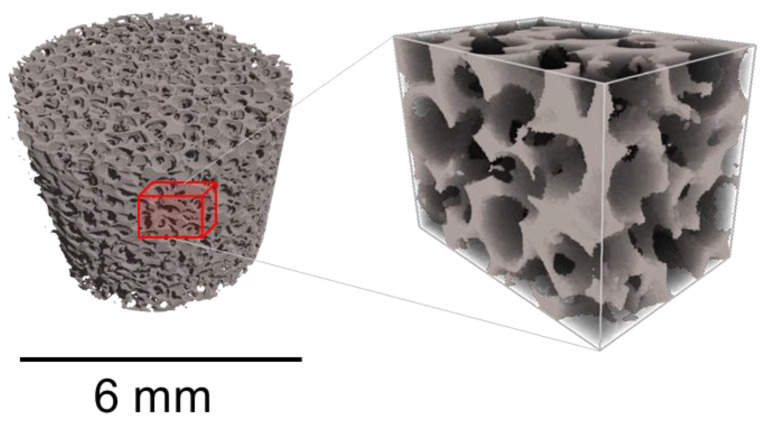
Segmented porous structure with extracted sub-volume (red prism) for the identification of cell type.

The X-ray tomography technique allowed detailed analysis of the three-dimensional foam structure, in particular determining the cell type within the UHMWPE foam microstructure illustrated in Figure 7. The produced UHMWPE foams were found to contain a mixture of open and close pores within cells with massive rigid walls. The shape of cells in these foams can be assumed to be quasi-spherical or elliptical in general (~95 vol. %), but a small fraction of quasi-prismatic cells was also found to be present due to crystallographic habit of the salt particles. Moreover, good agreement was found (relative error less than 10%) between two methods for porosity analysis based on the thresholding of images obtained by SEM (so-called “surface porosity”) and the segmentation of reconstructed tomography datasets (so-called “volume porosity”). Of course, the type, shape, and connectivity of cells as well as porosity of polymer structure are extremely dependent on the salt and UHMWPE powder particle size distribution and intermixing degree. For instance, in order to produce open-cell foam, larger salt particles should be adopted within a matrix made from small/medium UHMWPE particles.

### 3.2. DIC Analysis

**Figure 8:**
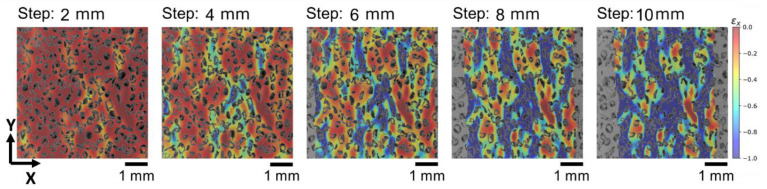
Strain distribution along *x*-axis for different compression stage of UHMWPE foam.

The representative result of the DIC analysis for in-SEM compression of UHMWPE foam is shown in Figure 8. The outcomes indicate a high degree of strain localization and indicate that the dominant mechanism of UHMWPE foam deformation under compression is indeed associated with microscale cell wall bending. The inhomogeneity of deformation in porous polymers requires careful high-resolution imaging and analysis and remains the subject of ongoing study. The DIC strain maps shown demonstrate the complexity and variability of strain at the scale of walls. It can be expected that thinner walls may undergo larger bending deformation. However, it is clear that this will also depend on the surrounding microstructure that controls the local moments and forces.

**Figure 9:**
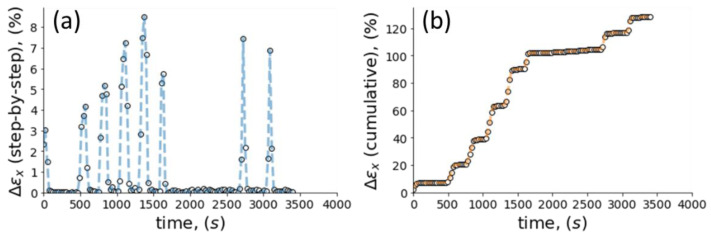
The relation of time-dependent $\Delta \varepsilon_{x}$ strain extracted from DIC maps: (**a**) step-by-step; (**b**) cumulative.

Stress relaxation testing is performed by keeping the specimen under applied permanent strain over a selected period of time. Strain maps of the difference between the current and reference states in the course of the relaxation process were obtained from DIC analysis (Figure 9). The results summarized in Table 1 show that strain along *x*-axis was changed by ~1% during the entire duration of the stress relaxation experiment.

### 3.3. Stress Relaxation Analysis

**Figure 10:**
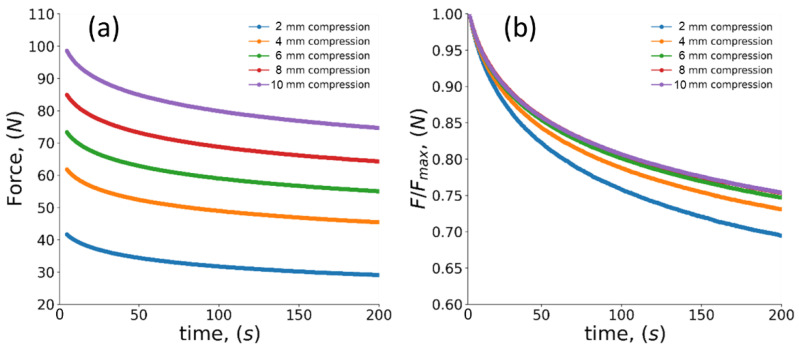
Force-dependent relaxation curves obtained during different compression step: (**a**) raw data, (**b**) normalized data.

The common relaxation curves recorded during the compression test at different stages are illustrated in Figure 10. Since it is difficult to distinguish changes between curves in Figure 10a, the curves were normalized with respect to the maximum force as shown in Figure 10b. In this case, it is possible to detect changes in decay of load during different compression steps, namely, the stress relaxation after the first compression by 2 mm is seen to unfold more slowly compared to other compression steps. The results for the two relaxation times measured under different compression steps for specimens with various porosity value are summarized in Table 2. Although small variation of stress relaxation time between solid material and foams was found, no statistically significant difference could be extracted for porous materials.

It is noted that the individual relaxation processes can be thought of as in-dependent, referring, for example, to the paper [26].

In general, polymeric materials have relaxation components longer than 10^4^ s. However, it was observed by in situ bending and release of porous UHMWPE bar inside SEM that structural elements of porous UHMWPE exhibited a fast relaxation mechanism. This effect is particularly apparent in Supplementary Materials Video S1.

**Figure 11:**
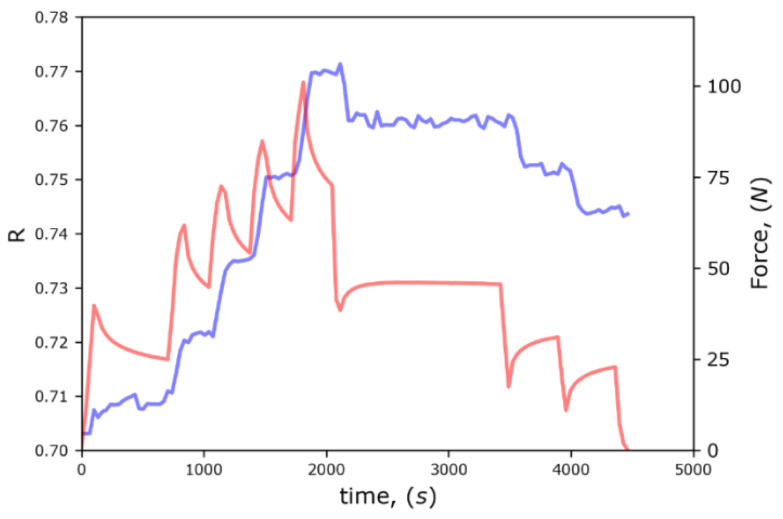
The correlation between apparent surface relative density $R_{S}$ (light blue color) and applied force (light red color) during compression.

The apparent surface relative density ($R_{S}$) changes during compression test as well as force value were plotted as a function of time and are shown in Figure 11. The main outcome of this result is that behavior of $R_{S}$ parameter (light blue color) has a positive correlation with the applied force (light red color). This confirms that the relative density is the principal parameter for the determination of the mechanical response (load) and stiffness. The segmentation method of 2D images has several constrains related to the appearance of subsurface structure inside pores, affecting the method precision. However, this was checked by gravimetric measurements, determining the mass and volume of composite before and after salt dissolution, and by X-ray tomography results. The three methods showed good agreement at different values of porosity. It was concluded that the segmentation method can be used for this analysis provided good quality images are employed and threshold is set properly based on cross-validation.

### 3.4. In Situ SAXS Tests

**Figure 12:**
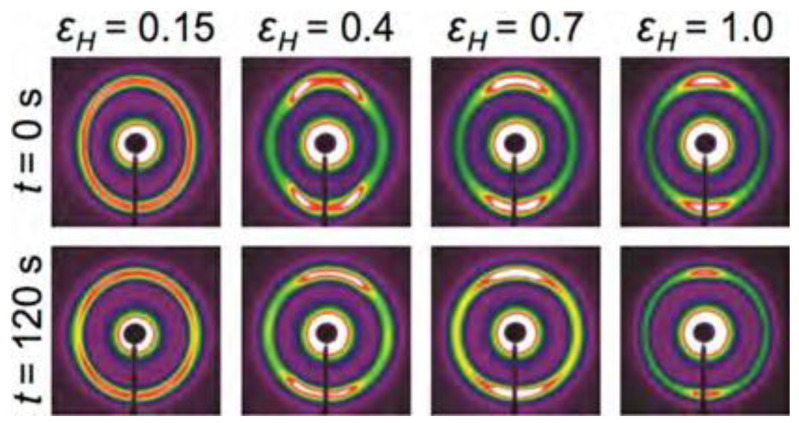
SAXS images that represent relaxation behavior (columns) from samples that were subjected to an extension rate $\dot{\varepsilon }$ = 0.01 s^−1^ to various values of Hencky strain ($\varepsilon_{H}$). Reprinted with permission from [27] (2014) *Macromolecules*, ACS Publications.

One of the hypotheses presented in the paper concerns the role of modification of molecular conformation in polymer relaxation. SAXS is known to be sensitive to this process at the nanoscale. This is illustrated in the time lapse SAXS-WAXS patterns taken at different applied strain levels as shown Figure 12 [27].

**Figure 13:**
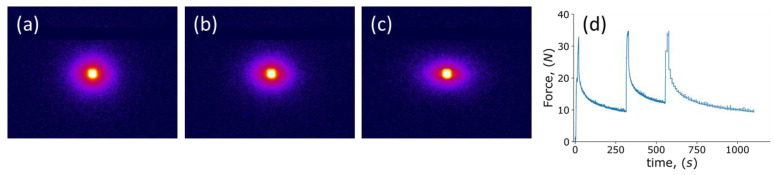
Results of in situ SAXS-tension experiment: (**a**–**c**) 2D SAXS images during in situ tension; (**d**) recorded *F(t)* curve.

The in situ SAXS data collected during the tension test are shown in Figure 13. The 2D SAXS patterns are shown in Figure 13a–c. The first scattering pattern shown reveals the isotropic nature of the material. Upon tensile deformation in the vertical direction, the sample molecular structure becomes elongated vertically, as reflected in the pattern shrinking in this direction, assuming the shape of an ellipse with the smaller vertical semi-axis. This reflects the fact that scattering is recorded in the reciprocal space, so that larger structural dimensions lead to smaller scattering angles. Simultaneously, the horizontal scattering angles increase, reflecting the shrinking of the scattering elements (crystalline-amorphous region interfaces) due to the Poisson effect. It is worth noting that for polymers the values of Poisson’s ratio are typically larger than those for metals and ceramics, approximately equal to 0.3 for UHMWPE.

Considering tension here was experimentally expedient (given the need to use of thin film of polymer as opposed to thick compression samples), but also provided a chance to compare the relaxation times found under these conditions with those from compression. Figure 13d illustrates the time-dependent force curve. Similar to the observation made for compressive loading, under tension, the relaxation times for UHMWPE do not appear to change during deformation. The two relaxation times were found to be equal to 10 s for the ‘short’ relaxation time and 150 s for the ‘long’ time, respectively. The ‘long’ relaxation time during tension shows close correspondence to that under compression, while the ‘short’ relaxation time differs by a factor of two. This is likely to be connected with the difference in the molecular accommodation processes that occur in these two modes. Given the importance of free volume for the relaxation process, it is concluded that somewhat shorter relaxation times under tension are associated with the increase of free volume (v.v., under compression the free volume is somewhat reduced).

In contrast to the elastic behavior that differs strikingly between monolithic and porous samples (see the section below devoted to stiffness), it is notable that the viscous response remains very similar between the two forms of material and is characterized by two relaxation times of tens and hundreds of seconds, respectively. This refers to finer sub-micron scales than those involved in the formation of the porous polymer. What remains to be investigated is the effect of material’s degree of crystallinity and supramolecular structure at the sub-micron scale. Understanding the underlying molecular relaxation mechanisms requires concerted effort involving theoretical and numerical modelling on the one hand (e.g., molecular dynamics calculations [28]), and advances in the experimental techniques on the other (i.e., AFM [29], SAXS [30], WAXS [31]).

**Figure 14:**
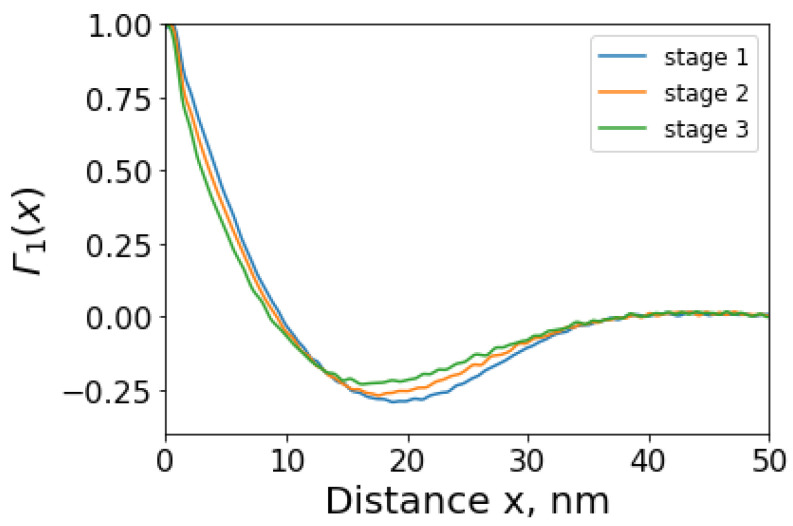
Electron density auto-correlation plot for the determination of semi-crystalline polymer structural parameters (“short” and “long” periods, etc.).

Structural parameters of semicrystalline polymers can be extracted from the analysis of SAXS patterns. Figure 14 illustrates the electron density auto-correlation plot that allows the determination of the so-called “average hard block thickness” of the fibril, i.e., stack of the crystalline lamellae in polymers. The average hard block thickness values lie close to the first minimum and are found by the intercepts between the downward tangent to the initial part of the plot and the horizontal line tangent to the first minimum. For the three elongation states, the plots shown give values of approximately 11.5 nm, 13.6 nm, and 15.9 nm respectively, reflecting the stretching effect of the imposed deformation. Detailed analysis of these parameters and their correlation with macroscopic strain represent a complex task [32,33,34] that requires separate further investigation.

### 3.5. Stiffness Evolution Analysis

Although there are multiple analytical approaches to property prediction in foam mechanics, all of them possess some limitations due to the assumptions made regarding the foam structure and its behavior during deformation. For instance, Ko [35] investigated the correlation between stiffness, network geometry and strut deformation mechanisms for low-density open-cell foams. They observed a change in the dominant deformation mechanisms during mechanical test from initial tension to latter bending. However, the most popular model was developed by Gibson and Ashby [36]. In this model, they assumed bending to be the key mechanism of strut deformation and defined the foam unit cell as a cube that can incorporate a thin membrane on each face (closed-cell) or be without it (open-cell). Amongst other aspects, this allows distinguishing between low- and high-density foams.

The mechanical properties of different foams were studied in many papers [37,38]. It was declared that density (or relative density compared to bulk material) is the primary parameter that affects porous structure behavior. The relative density parameter is defined here as $R=\frac{\rho_{f}}{\rho_{s}}$, where $\rho_{f}$ is the foam density and $\rho_{s}$ is a density of the solid material. According to the Gibson and Ashby [36] model, cell edges of closed-cell foams both bend and extend or contact, while the membranes stretch, contributing to the wall stiffness and elastic moduli of the structure. Provided the membranes do not rupture, the pressure variation of entrapped air also contributes to the overall stiffness. To describe the dependence of the elastic modulus on the overall modulus of a porous polymer, we propose a formula that includes three contributions as follows

(3) $$\frac{E_{f}}{E_{s}}=\phi^{2}R^{2}+\left(1-\phi \right)R+\frac{p_{0}}{E_{s}\left(1-R\right)},$$

where $E_{f}$ is Young’s modulus of the foam (poroid),

$E_{s}$ is Young’s modulus of the solid material,
ϕ is the fraction of solid contained in the cell edges—$\phi \in \left[0.6;0.8\right].$

**Figure 15:**
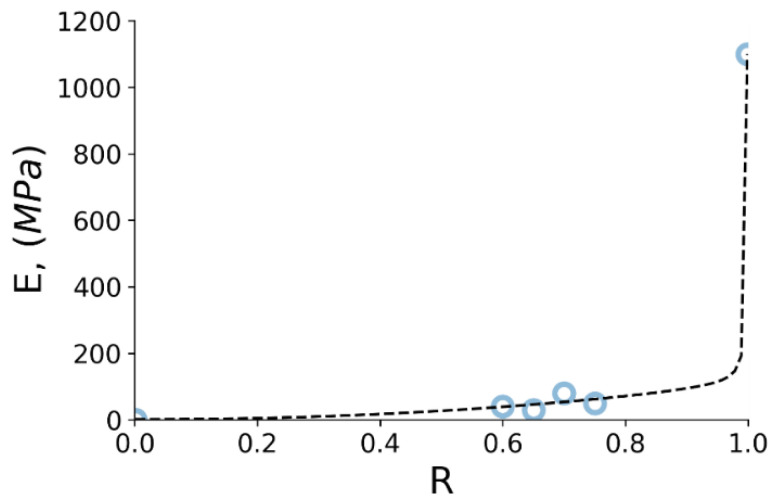
Young’s modulus over relative density relationship for produced UHMWPE foams. Blue open circles correspond to the UHMWPE sponges and solid samples studied.

The result of applying the above equation to the obtained data is shown in Figure 15. We assume the following boundary conditions:

(1) The material with relative density equal to zero has null elastic modulus.
(2) The solid UHMWPE has Young’s modulus of 1100 MPa that was determined from the experiment.

The fitted function in Figure 15 shows very steep variation close to R=1 and would benefit from more detailed calibration for this range of values. However, the preparation of a specimen with porosity in the range 5–20% corresponding to the R values 0.95–0.8 is an extremely challenging task from the practical point of view, at least using the procedures used in this study.

## 4. Conclusions

(1) Stress relaxation of solid (R = 1) and porous (R = 0.60/0.65/0.70/0.75) UHMWPE was studied.
(2) Statistically significant conclusions could be drawn from the comparison between experiment and theory. The obtained relaxation curves were fitted with simple two-term Prony series where two relaxation times were extracted. It was postulated that the observed times are connected with molecular (physical cross-links and entanglements) and structural (recovery of cell walls, trusses and nodes in porous structure) relaxation mechanisms, respectively. The two times were found to be approximately equal to 20 s and 160 s.
(3) Porous structures, i.e., geometrical characteristics of the pore space, were investigated by X-ray tomography. The results showed a mixture of open and close pores within cells with massive solid walls.
(4) The relaxation times are almost independent of the porosity and the apparent compression strain.
(5) The apparent stiffness of the porous polymer was matched with a three-term expression derived on the basis of the Gibson and Ashby model.
(6) Further investigations are needed to study the temperature dependence of the relaxation parameters.

## Figures and Tables

**Table 1:** Strain changes along *x*-axis during stress relaxation process.

Time, s	$\Delta \varepsilon_{x},\;\%\;(\mathrm{Step}\text{-}\mathrm{by}\text{-}\mathrm{Step})$	$\Delta \varepsilon_{x},\;\%\;(\mathrm{Cumulative})$
0	-	-
75	0.06	0.06
150	0.04	0.10
225	0.07	0.17
300	0.55	0.72

**Table 2:** The results of measured relaxation times for specimens with different porosity.

$\text{‘}\mathrm{Low}\text{’}\;\mathrm{Force}\;\mathrm{Component}\;(\tau_{1})$		Porosity, %			
		0	25	30	40
Compression stage	2 mm	178 ± 8	145 ± 13	145 ± 8	149 ± 6
	4 mm	187 ± 7	142 ± 10	152 ± 8	162 ± 6
	6 mm	183 ± 6	148 ± 11	153 ± 7	159 ± 6
	8 mm	189 ± 6	148 ± 10	152 ± 7	162 ± 5
**‘High’ force component (** $\tau_{2}$ **)**		**Porosity, %**			
		**0**	**25**	**30**	**40**
Compression stage	2 mm	23 ± 1	25 ± 2	17 ± 1	18 ± 1
	4 mm	24 ± 1	16 ± 2	17 ± 1	20 ± 1
	6 mm	22 ± 1	18 ± 2	18 ± 1	19 ± 1
	8 mm	23 ± 1	17 ± 2	18 ± 1	19 ± 1

## Data Availability

The data presented in this study are available on request from the corresponding author.

## Supplementary Materials

The following supporting information can be downloaded at: https://www.mdpi.com/article/10.3390/polym14245374/s1, Video S1: In situ bending and release of porous UHMWPE bar inside SEM.
